# Surgical treatment of intracystic carcinoma of the breast

**DOI:** 10.1186/1477-7819-9-116

**Published:** 2011-10-04

**Authors:** Masahiro Kitada, Satoshi Hayashi, Yoshinari Matsuda, Kazuhiro Sato, Naoyuki Miyokawa, Tadahiro Sasajima

**Affiliations:** 1 Department of Surgery, Asahikawa Medical University, Asahikawa, Hokkaido, Japan; 2 Department of Clinical Pathology, Asahikawa Medical University, Asahikawa, Hokkaido, Japan

## Abstract

**Background:**

Intracystic carcinoma of the breast is a type of breast cancer with favorable prognosis where cancer arises from the cystic wall. However, it is a relatively rare disease, and no general consensus has been reached on its definition, including pathogenesis, extramural invasion, and lymph node metastasis.

**Methods:**

Six patients who underwent surgery at the Department of Surgery at Asahikawa Medical University are presented. In each patient, background factors, diagnosis, surgery, pathological diagnosis, and prognosis were investigated.

**Results:**

Fine needle aspiration showed class V disease in three patients and class III disease in the other three, and lumpectomy was performed for class III patients. Three patients underwent breast-conserving surgery While extramural invasion was seen in three patients, lymph node metastasis was absent in all patients.

**Conclusion:**

When it is difficult to diagnose intracystic carcinoma of the breast by fine needle aspiration, active lumpectomy is necessary. Because extramural invasion and lymph node metastasis have been reported, it is necessary to carefully determine the range of excision and rationally perform lymph node dissection, such as sentinel node biopsy.

## Background

Intracystic carcinoma of the breast is a type of breast cancer with favorable prognosis where cancer arises from the cystic wall. It is a relatively rare disease, and includes ductal carcinoma in situ according to the Japanese Society for Breast Cancer.

However intracystic carcinoma is difficult to diagnose than common breast carcinoma, no general consensus has been reached on its definition, including pathogenesis, extramural invasion, and lymph node metastasis. Six patients with this condition were clinically investigated and a literature review was conducted.

## Methods

Of 1160 breast cancer patients who underwent surgery at Asahikawa Medical University Hospital from January 2001 to March 2010 subjects were six patients who were histopathologically diagnosed with intracystic carcinoma of the breast (0.5%). In each patient, background factors, diagnosis, surgery, pathological diagnosis, and prognosis were investigated.

## Results

### 1. Background factors and preoperative diagnosis (Table 1)

**Table 1:** Background factors and preoperative diagnosis

	Age	Sex	Chief complain	Duration of symptom	Localization	Size of tumor	Liquid status	Cytology
1	65	♂	Breast mass	1 Y	E	2.5 cm	brown	3a

2	53	♀	Breast mass	1 M	A	2.5 cm	bloody	3a

3	81	♀	Breast mass	1 M	C	3.0 cm	bloody	5

4	80	♀	Breast mass	3 M	E	6.0 cm	bloody	3b

5	89	♀	Breast mass	2 M	BE	3.5 cm	bloody	5

6	74	♀	Breast mass	1 M	ED	2.5 cm	bloody	3

Localization: A; The inner upper part, B; The inner lower part, C; The outside upper part, D; The outside lower part, E; The under areola part

Cytology was diagnosed by the Papanicolous classification.

Patients were one man and five women with an average age of 73.3 years (range: 53 - 89 years); the average age of patients with other forms of breast cancer in comparable stages was lower at 53.6 years (range: 28 - 86 years). The main complaint for all patients was a breast lump, and none reported bloody discharge from the nipple. The length of time from first symptoms to diagnosis was relatively short at 1 - 3 months for the five women, but longer at 1 year for the man. The male patient was not aware of the existence of breast cancer in men, and he visited the hospital only after the tumor had increased in size.

One patient had undergone surgery for breast cancer in the contralateral breast 18 years previously. Mammography (MMG) showed a shape of mass, and ultrasonography (US) confirmed solid component with intracystic growth and most cases showed that solid components were variable, regular or irregular in shape. Fine needle aspiration cytology (FNA) revealed light brown cystic fluid in one patient and bloody fluid in the remaining five. It was diagnosed by the Papanicolous classification. Cytologic class was class V in three patients and class III in the other three. Lumpectomy was performed on the three patients with class III cytology.

### 2. Operation and pathology (Table 2)

**Table 2:** Operation method, pathological findings, receptor status, treatment and outcome

No	Operative method	P-stage	Grade	Extramural invasion	Vascular invasion	Receptor status	Medical treatment
1	Bt+Ax	0	I	-	ly(-) v(-)	ER(1+) PgR(2+) HER2(3+)	Endocrine therapy (TAM)

2	Bp+Ax	I	I	+(IDC)	ly(-) v(-)	ER(-) PgR(-) HER2(-)	-

3	Bp	0	I	-	ly(-) v(-)	ER(3+) PgR(3+) HER2(2+)	Endocrine therapy (AI)

4	Bt+SN	I	II	+(IDC)	ly(-) v(-)	ER(3+) PgR(-) HER2(-)	Endocrine therapy (AI)

5	Bt+SN	I	I	+(DCIS)	ly(-) v(-)	ER(3+) PgR(3+) HER2(1+)	Endocrine therapy (AI)

6	Bp+SN	I	I	+(IDC)	ly(-) v(+)	ER(3+) PgR(3+) HER2(-)	Endocrine therapy (AI)

Bt: Breast total resection, Bp: Breast partial resection, Ax: Axillal lymph node resection, SN: sentinel node biopsy, IDC: Invasive Ductal Carcinoma, DCIS: Ductal carcinoma in situ, ly: lynph duct invasion, v: vessel invasion, ER: Esterogen reseptor, PgR: Progesteron reseptor, HER2: Human epidermal growth factor receptor type2, TAM: Tamoxifen, AI: Aromatase inhibitor.

Three patients underwent breast-conserving surgery and the other three underwent mastectomy. With regard to lymph nodes, axillary lymph node dissection was performed on two patients and sentinel lymph node biopsy on three patients; lymph node dissection was not performed on one patient. Histopathological analyses showed intracystic papillary carcinoma in all patients, and lymph node metastasis was not seen. Postoperative staging was either 0 or I. A concurrent malignant extramural invasion was seen in four patients (DCIS in one patient, and invasive ductal breast cancer in the other three). One patient had multiple small invasive cancerous lesions around the tumor. One patient tested negative for estrogen receptors and the remaining five tested positive, and hormone therapy was performed for those who tested positive (Tamoxifen (TAM) for the male patient and aromatase inhibitor (AI) for the postmenopausal women). In the three patients who underwent breast-conserving surgery, radiotherapy was performed on the remaining breast. At present, no recurrences have been detected and all patients remain alive without cancer.

## Discussion

Intracystic carcinoma of the breast was first reported by Brodie and colleagues [1], and it is a relatively rare disease, accounting for only 0.5 to 1.9% of all breast cancers [2,3]. According to the general rules for clinical and pathological recording of breast cancer, a lesion localized in a cyst is defined as noninvasive intracystic carcinoma of the breast, but there is no mention of extramural invasion [4]. However, because it is not easy to prove whether a tumor develops in a benign cyst or a cyst is formed due to the secondary changes associated with tumor necrosis, there is no general consensus on definition of this lesion, including pathogenesis, invasion, and lymph node metastasis. The present report is significant because breast cancer in which a lesion existed within the cystic wall was investigated as intracystic carcinoma of the breast.

Patients with intracystic carcinoma of the breast tend to be older than patients with other forms of breast cancer. In our study, the average age was 73.4 years, which is about 20 years older than the average age of other breast cancer patients (53.6 years). The present study also included one male patient. Since breast cancer in men accounts for only about 1% of all breast cancers, the incidence of intracystic carcinoma of the breast in men appears to be higherand and there is equal to or more than 5% of report [5,6].

Most patients with intracystic carcinoma of the breast have symptoms such as a palpable mass and bloody discharge, and it is relatively easy to delineate a timorous lesion in the cyst by US. Moreover, cyst fluid is characteristically bloody. FNA reveals class IV/V cytology in 63 - 65%, but malignant cells are not seen in some cases [7].

We perform FNA on tumorous lesions under US guidance, and if intracystic carcinoma is suspected, cystic fluid is bloody, or FNA reveals a cytologic grade of class III or higher, we actively perform lumpectomy to confirm diagnosis. The level of CEA in the cystic fluid has also been measured, with levels ≥ 400 ng/ml reported to indicate malignancy [8]. Because many examples not to be taking MRI imaging in our cases, it wasn't possible to have entered reviewing. There is a report which is useful for the diagnosis of Intracystic carcinoma [9], and it wants to review it in the future.

With regard to therapy, breast-conserving surgery appears to be indicated, but there have been some reports of extramural invasion, intraductal progression, and axillary lymph node metastasis including micrometastases [10,11]. It is therefore necessary to consider the indications for breast-conserving surgery, as in other breast cancers. In the present study, extramural invasion was seen in four of the six patients. Patients with extramural invasion are at increased risk for lymph node metastasis, and axillary lymph node dissection was therefore performed in the past in this situation. However, in recent years, sentinel lymph node biopsy has become a standard procedure, and in intracystic carcinoma of the breast, sentinel lymph node biopsy should be considered for preoperative class V diagnosis. When lumpectomy is performed, sentinel lymph node biopsy is difficult, and in such a case, a reductive operation such as lymph node sampling is needed. With regard to postoperative adjunctive therapy, the same standards as for other types of breast cancer are employed.

Clinicopathological analyses were performed on six patients with intracystic carcinoma of the breast. The results suggested that lumpectomy should be actively performed when cystic fluid is bloody and FNA reveals class III cytology. Since there have been reports of extramural invasion and lymph node metastasis, it will be necessary to carefully determine the extent of resection based on rapid pathological analysis and to perform rational lymph node diagnosis, such as sentinel lymph node biopsy.

## Conclusion

When it is difficult to diagnose intracystic carcinoma of the breast by fine needle aspiration, active lumpectomy is necessary. Because extramural invasion and lymph node metastasis have been reported, it is necessary to carefully determine the range of excision and rationally perform lymph node dissection, such as sentinel node biopsy.

## Consent statement

Informed consent was obtained from the patients for publication of this research and accompanying images. A copy of the written consent is available for review by the Editor-in-Chief of this journal.

## Competing interests

The authors declare that they have no competing interests.

## Authors' contributions

MK have operated this case and analyzed all data. SH and YM, KS did the assistant of the operation. NM diagnosed h the pathology of this case.

TS is the professor of the surgical science and had a guide.

All authors read and approved the final manuscript.
